# Valsartan Upregulates Kir2.1 in Rats Suffering from Myocardial Infarction via Casein Kinase 2

**DOI:** 10.1007/s10557-015-6598-1

**Published:** 2015-06-23

**Authors:** Xinran Li, Hesheng Hu, Ye Wang, Mei Xue, Xiaolu Li, Wenjuan Cheng, Yongli Xuan, Jie Yin, Na Yang, Suhua Yan

**Affiliations:** School of Medicine, Shandong University, Ji’nan, Shandong China; Department of Cardiology, Shandong Provincial Qianfoshan Hospital, No. 16766 Jingshi Road, Jinan, 250014 Shandong Province China

**Keywords:** Myocardial infarction, CK2, Kir2.1, Valsartan, Rat

## Abstract

**Purpose:**

Myocardial infarction (MI) results in an increased susceptibility to ventricular arrhythmias, due in part to decreased inward-rectifier K+ current (IK1), which is mediated primarily by the Kir2.1 protein. The use of renin-angiotensin-aldosterone system antagonists is associated with a reduced incidence of ventricular arrhythmias. Casein kinase 2 (CK2) binds and phosphorylates SP1, a transcription factor of KCNJ2 that encodes Kir2.1. Whether valsartan represses CK2 activation to ameliorate IK1 remodeling following MI remains unclear.

**Methods:**

Wistar rats suffering from MI received either valsartan or saline for 7 days. The protein levels of CK2 and Kir2.1 were each detected via a Western blot analysis. The mRNA levels of CK2 and Kir2.1 were each examined via quantitative real-time PCR.

**Results:**

CK2 expression was higher at the infarct border; and was accompanied by a depressed *IK1/Kir2.1* protein level. Additionally, CK2 overexpression suppressed *KCNJ2/Kir2.1* expression*.* By contrast, CK2 inhibition enhanced *KCNJ2/Kir2.1* expression, establishing that CK2 regulates *KCNJ2* expression. Among the rats suffering from MI, valsartan reduced CK2 expression and increased Kir2.1 expression compared with the rats that received saline treatment. In vitro, hypoxia increased CK2 expression and valsartan inhibited CK2 expression. The over-expression of CK2 in cells treated with valsartan abrogated its beneficial effect on KCNJ2/Kir2.1.

**Conclusions:**

AT1 receptor antagonist valsartan reduces CK2 activation, increases Kir2.1 expression and thereby ameliorates IK1 remodeling after MI in the rat model.

## Introduction

Ventricular arrhythmias following myocardial infarction (MI) remain a major cause of mortality [1]. Numerous studies have confirmed that decreased inward-rectifier K+ current (IK1), along with the decreased expression of KCNJ2 mRNA and its encoded Kir2.1 protein, is a prominent feature of ventricular electrical remodeling following MI [2–4]. IK1 is the primary K+ current that maintains resting membrane potential, controls cardiac excitability and modulates both late-phase repolarization and action potential duration (APD) in cardiac cells. Furthermore, IK1 plays an important role in cardiac excitability and arrhythmogenesis and is a promising target for new antiarrhythmic approaches [5].

The mechanism underlying IK1 dysregulation following MI primarily involves intracellular signaling pathways. However, the gene regulation of these pathways is poorly understood. Recent studies have discovered that CK2 is associated with several diseases, such as cardiac hypertrophy [6], and is also involved in ion channel regulation [7, 8]. Additionally, several studies have demonstrated that CK2 binds to and induces the phosphorylation of transcription factor SP1 serine, resulting in the suppression of gene expression [9, 10]. SP1 is also an important transcription factor for KCNJ2 [11]. Therefore, we hypothesized that CK2 regulates KCNJ2/Kir2.1/IK1 expression via CK2.

The use of renin-angiotensin-aldosterone system (RAAS) antagonists is associated with a reduced incidence of malignant arrhythmias [12]. Therefore, we used a rat model of MI to determine whether the angiotensin type 1 receptor antagonist, valsartan, downregulates CK2 and increases the expression of Kir2.1 following MI.

## Materials and Methods

### Ethics Statement

The animals were handled and all procedures were performed in accordance with the regulations of the *Guide for the Care and Use of Laboratory Animals,* published by the United States National Institutes of Health (NIH publication no. 85–23, revised 1996) and approved by the Animal Care and Use Committee of Shandong University.

### Cell Culture

The H9c2 (Wistar rat embryonic ventricle) cell line used in this study was purchased from ATCC (Zhongyuan Ltd., Beijing, China) and cultured in DMEM.

### Cardiomyocyte Isolation and Primary Cell Culture

The enzymatic dispersion techniques used to isolate single ventricular myocytes from neonatal Wistar rats have been described previously in detail [13]. Briefly, 1- to 3-day-old rats were decapitated, and their hearts were removed in a sterile manner. The apex of each heart was dissected, minced, and trypsinized at 37 °C for 10 min. Dissociated cells were plated in 6-well plates in DMEM (Invitrogen) containing 10 % FBS, and the nonadherent cardiomyocytes were removed. The cells (1–2 *10^5^/well) were seeded onto a 6-well plate for further experiments. This procedure yielded cultures with 80 ± 10 % myocytes, as assessed via the microscopic observation of the cells.

### Drug Treatment

The CK2 inhibitor, 4,5,6,7-tetrabromo-2-azabenzimidazole (TBB), was purchased from the Sigma-Aldrich Company (Sigma, St. Louis, MO, USA). TBB was dissolved in 100 % dimethylsulphoxide (DMSO; Sigma) to make a stock solution of 10 mM, which was then diluted in culture medium to obtain the desired concentration of 100 μM [14, 15]. Untreated cells were incubated in culture medium without any additives. The cells were treated either with or without TBB for 48 h.

CoCl_2_ (300 μM) (Sigma, St. Louis, MO, USA) and valsartan (20 μM) (Novartis Pharma AG, Basle, Switzerland) were prepared in double distilled water, diluted with culture media and cultured for 48 h. The doses of both CoCl_2_ and valsartan were similar to those used in previous studies [16, 17].

### Transfection Procedures

To achieve the transient overexpression of CK2, neonatal rat ventricular myocytes and H9c2 cells were transfected with pcDNA6-CK2α at a dose of 2.0 μg/mL, using *N*-[1(2,3dioleoyloxyl)propyl]-*N,N,N*-trimethylammoniummethylsulfate (DOTAP) for a period of 24 h, as was performed in previous studies [18, 19]. The cells were transfected for 48 h.

### MI Model

Male Wistar rats (8 weeks-of-age, 250–300 g); provided by the Animal Facilities of Shandong University, China; were anesthetized with an intraperitoneal injection of 3 % sodium pentobarbital (40 mg/kg; Sigma-Aldrich, St. Louis, Mo., USA). The animals underwent a thoracotomy and pericardiotomy, and the left anterior descending coronary artery was ligated to induce an MI as previously described [20]. The sham rats (*n* = 10) underwent a thoracotomy and pericardiotomy without coronary artery ligation. The MI rats received either oral valsartan (10 mg/kg/day once a day, *n* = 10) or an equivalent volume of saline (*n* = 10) for 7 days, beginning on the day after the operation. The dose of valsartan was similar to that used in previous studies [21].

### Heart Tissue Preparation

**Fig. 1:**
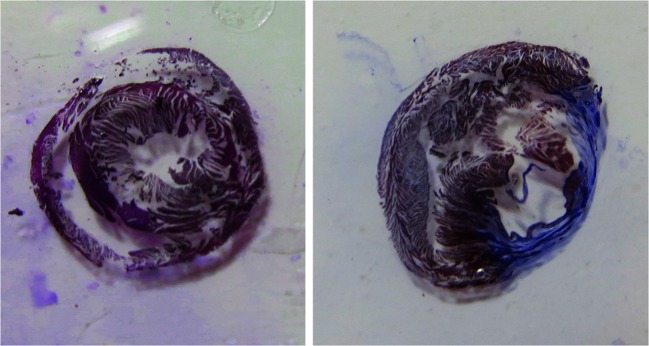
Representative histologic image of the heart stained with Masson’s Trichrome. Myocytes are *red* and fibrotic tissues are *blue. Left*: after the sham surgury. *Right*: after the MI surgury

The heart was rapidly excised, sliced along the edges of the infarction, and dissected along the infarct border. The middle portion included the entirety of the infarcted myocardium and was immersed in 10 % formalin for 24 h, embedded in paraffin, cut into 10-μm sections and stained with Masson’s trichrome (Fig. 1). We sampled the corresponding heart positions in the sham rats.

### qPCR Analysis

We conducted qPCR experiment as previously described [22, 23]. Briefly, total RNA was extracted from the rat hearts, the H9c2 cells and the primary ventricular myocytes using Trizol (Invitrogen, Carlsbad, Calif., USA), before being reverse-transcribed using a PrimeScript RT reagent kit (TaKaRa Biotechnology, Dalian, China). Real-time PCR was performed using a SYBR Premix Ex Taq (TaKaRa) in a Master-cycler EP Realplex detection system (Roche, USA), according to the manufacturer’s instructions. Each measurement was performed in triplicate. The data analysis was performed using the 2–△△CT method [24]. The primer sequences were as follows: Kir2.1, forward: 5′- TGCCCGATTGCTGTTTTC-3′, and reverse: 5′- GGCTGTCTTCGTCTATTT- 3′; CK2, forward: 5′- GTGGTGGAATGGGGAAATCAAGA -3′, and reverse: 5′- GTATCGGGAAGCAACTCGGACAT- 3′; β-actin, forward: 5′- ACCACAGTCCATGCCATCAC-3′, and reverse: 5′-TCCACCACCCTGTTGCTGTA-3′.

### Western Blot Analysis

Protein samples were extracted from the infarct border zones of the rat hearts, from cultured neonatal rat ventricular myocytes and from H9c2 cells utilizing procedures described in detail elsewhere [17]. Nuclear and cytoplasmic proteins were isolated using a Nuclear and Cytoplasmic Protein Extraction Kit (Beyotime Institute of Biotechnology, Jiangsu, China). Protein content was determined using the BCA Protein Assay Kit (Beyotime).

Equal amounts of protein samples were fractionated using SDS-PAGE (6–12 % polyacrylamide gels) and transferred onto polyvinylidene difluoride membranes (Bio-Rad, Richmond, Va., USA), which were blocked with 10 % bovine serum albumin (Sigma-Aldrich) and incubated overnight at 4 °C with either rabbit anti-Kir2.1 (1:1500; Epitomics, Burlingame, Calif., USA) or rabbit anti-CK2alpha (1:1000, Abcam, Cambridge, MA, USA), followed by incubation with horseradish peroxidase-conjugated secondary goat anti-rabbit antibodies (1:10,000; Zhong Shan Golden Bridge Biotechnology). The relative expression levels of the target proteins were normalized using an anti-β-actin (1:5000; Proteintech, Chicago, USA) antibody. Imaging was performed using the FluroChem E Imager (ProteinSimple, Santa Clara, Calif., USA), with an enhanced chemiluminescence system (Millipore), and signal intensities were quantified using Image J software. The final results are expressed as fold changes by normalizing the data to the control values.

### Whole-Cell Patch-Clamp Recording

Patch-clamp techniques were applied to cultured neonatal rat ventricular myocytes transfected with miRNA or AMOs or negative control constructs. The pipettes used for the patch electrodes had tip resistances of 2 to 3 MΩ when filled with pipette solution. The cells were placed in a 1 ml chamber mounted on an inverted microscope (DMI3000 B; LEICA) and perfused with Tyrode’s solution. Whole-cell recording was performed using a patch EPC10 single amplifier (HEKA Instruments). The signals were filtered at 1 kHz, and the data were acquired via A/D conversion (LIH1600; HEKA Instruments). The ion currents were recorded in the whole-cell voltage-clamp mode. For the recordings of *IK1,* the pipette solution contained 130 mM KCl, 0.4 mM Na-GTP, 3 mM Mg-ATP, 0.5 mM EGTA, and 25 mM HEPES (pH 7.2 with KOH); the external Tyrode’s solution contained 135 mM NaCl, 4 mM KCl, 1.8 mM CaCl2, 1 mM MgCl2, 2 mM HEPES, and 11 mM dextrose (pH 7.4 with NaOH). CoCl_2_ (0.1 μM) and tetrodotoxin (10 μM) were both included to inhibit *IcaL* and *INa,* respectively*.* The experiments were conducted at room temperature. Series resistance and capacitance were compensated, and leak currents were subtracted. Cells with considerable leak currents were removed from the analysis. The data were collected using an IBM-compatible computer and analyzed using PatchMaster.

*IK1* was recorded with 200-ms square-wave pulses at voltages ranging from −120 mV to 0 mV with a holding potential of −80 mV [25, 26]. Individual currents were normalized to the membrane capacity to control for differences in cell size and are expressed as current densities (pA/pF).

### Electrophoretic Mobility Shift Assay (EMSA)

An EMSA was carried out as described previously [10]. The sequences of the oligonucleotides used for the EMSA were as follows: −31/+8, 5′-GTCACTTAAACAGCTGTGCAGTGGAAACAGTGTCAG-3′ and 5′-AGTCTGACACTGTTTCCACTGCACAGCTGTTTAAGT-3′; +9/+49, 5′-CTCGATTTCTCCTCCTACTCCTCCTCCGAGGAATTCT-3′ and 5′-GGGCAGAATTCCTCGGAGGAGGAGTAGGAGGAGAAAT-3′; +46/+90, 5′-GCCCCCTGTAACTGTTCTGCCCTCCCCTTTAAAGGTTGACTT-3′ and 5′-GGCAAGTCAACCTTTAAAGGGGAGGGCAGAACAGTTACAGGG-3′; +90/+118, 5′-GCCCTACGGCGCTCCACCGCGCTCCAGT-3′ and 5′-AGGACTGGAGCGCGGTGGAGCGCCGTAG-3′; +119/+160, 5′-CTTGCGCCTCCTGCTCAACCCGCTCCTGACTGCCCACGC-3′ and 5′-GCGGCGTGGGCAGTCAGGAGCGGGTTGAGCAGGAGGCG-3′; and +159/+195, 5′-CGCGTAGTTCCAGCAGCAAAGCAGAAGGGTGCA-3′ and 5′-CCGGTGCACCCTTCTGCTTTGCTGCTGGAACTA-3′.

Nuclear protein extracts were prepared using a commercially available kit (Viagene Biotechnology, Jiangsu, China). The EMSA involved the use of a nonradioactive EMSA kit (Viagene). Briefly, equal amounts of nuclear protein were incubated with poly dI:dC for 20 min at room temperature in binding reaction buffer. The specificity of the binding was examined via competition with an unlabeled oligonucleotide. The DNA-protein complexes were resolved on a 6.5 % polyacrylamide gel preelectrophoresed in 0.25 × Tris borate/EDTA at 120 V for 1 h. The gel was subsequently transferred onto a positively charged nylon membrane. The transferred DNA was cross-linked to the membrane and detected using horse- radish peroxidase-conjugated streptavidin.

### Data Analysis

The statistical analysis was performed using SPSS 10.5 software. The data are presented as the means ± standard deviations (SDs). The differences among multiple groups were assessed using a one-way analysis of variance (ANOVA), and a Tukey’s post-hoc test was used to evaluate the significance of the differences between 2 groups. A two-tailed *P* < 0.05 was indicative of a statistically significant difference. The number of rats or cells for each group is 10.

## Results

### The Dysregulation of CK2 and KCNJ2/Kir2.1 in the MI Rats

**Fig. 2:**
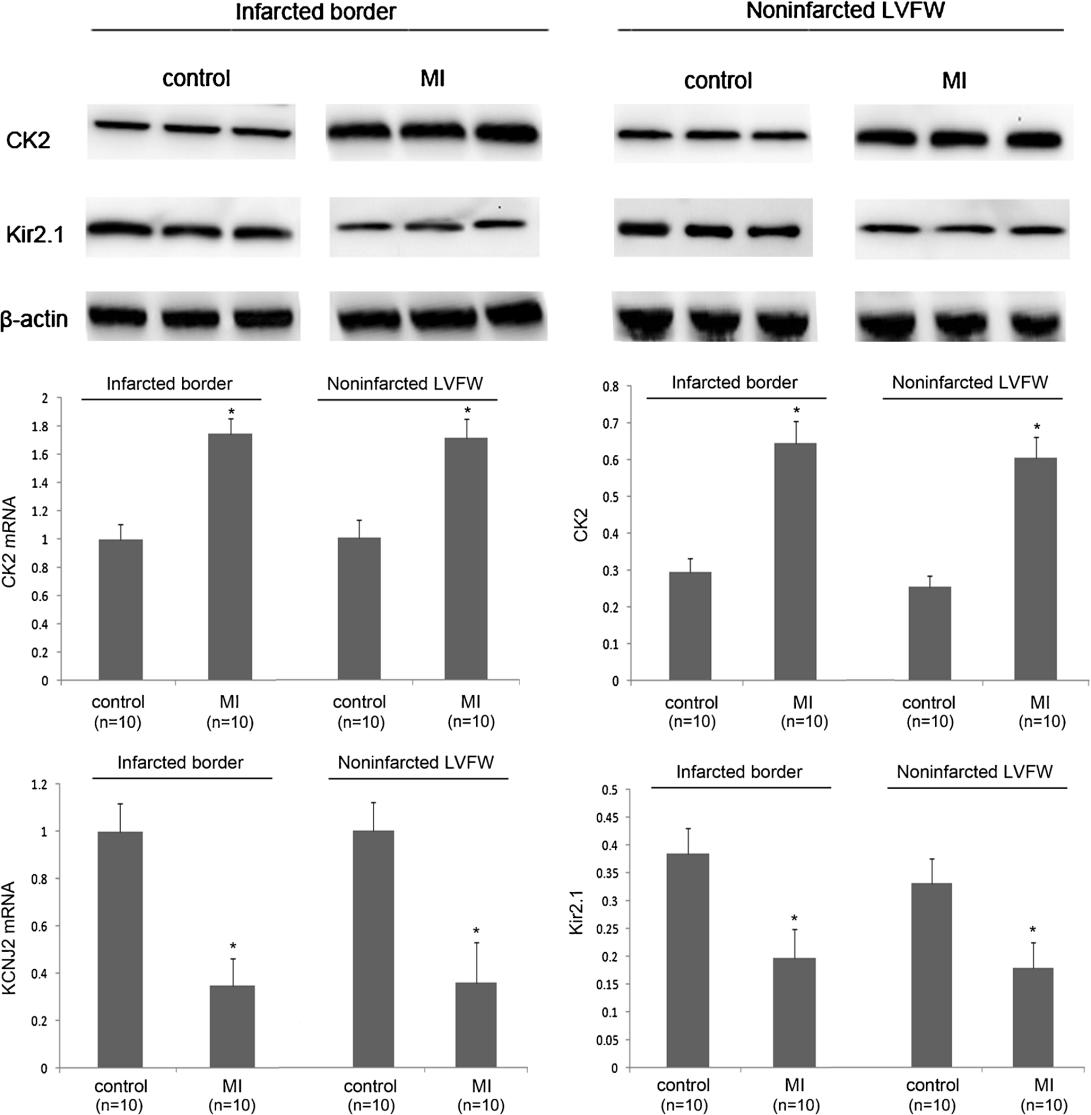
The upregulation of CK2 and the downregulation of KCNJ2/Kir2.1 in MI rats. A qPCR analysis and a Western blot analysis demonstrating the significant upregulation of CK2 and the downregulation of Kir2.1 in ventricular myocytes at a ventricular infarct border in a rat MI model. Similar results showing in noninfarcted LVFW of MI rats. *P < 0.05 vs. control; *n* = 10/group. Values are expressed as the means ± SDs

In an effort to determine the role of CK2 in acute myocardial infarction (AMI), we found that CK2 was significantly upregulated. Consistent with the findings of previous studies [27–29], we found that Kir2.1 protein expression was downregulated in MI rats (Fig. 2). Besides, the *KCNJ2* mRNA expression was also downregulated during the healing phase after AMI, suggesting that the regulation of Kir2.1 protein at 7 days after MI is not only on translation but also on mRNA level. These results are consistent with the hypothesis that CK2 contributes to *KCNJ2* dysregulation in the setting of MI, a possibility that we elected to test directly.

### The Validation of KCNJ2 as a Target for CK2

**Fig. 3:**
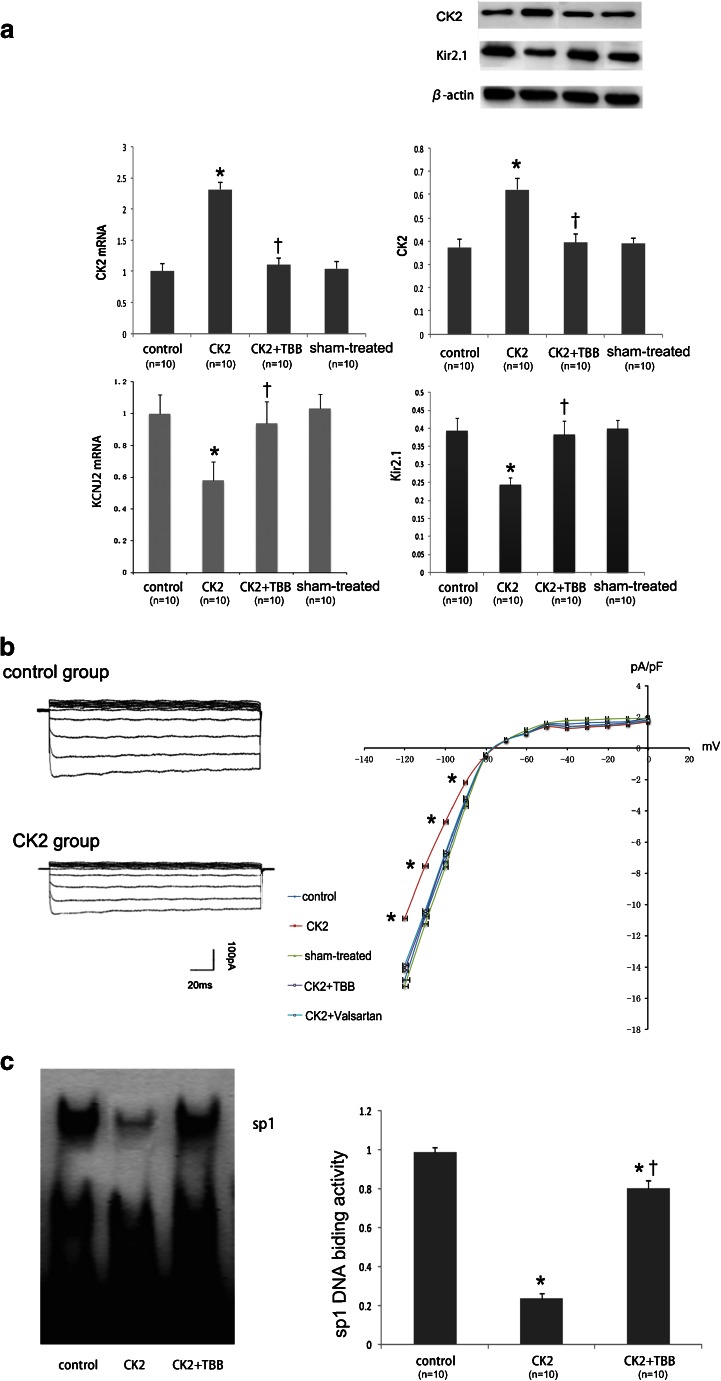
The regulation of Kir2.1 expression by CK2. **a** A qPCR analysis and a Western blot analysis demonstrating the CK2 level after transfection (*n* = 10) and inhibition by TBB (*n* = 10) and the effects of CK2 (*n* = 10) and its inhibition (TBB; *n* = 10) on Kir2.1 protein expression in H9c2 rat ventricular cells. *P < 0.05 vs. control; ^†^ *P* < 0.05 vs. CK2 alone. **b** IK1 density in cultured neonatal rat ventricular cardiomyocytes. IK1 was elicited by 200-ms pulses at the indicated voltages. *P < 0.05 vs. control; *n* = 10/group. **c** Autoradiograms and the EMSA quantification of Sp1 DNA-binding activity in H9c2 rat ventricular cells. The data are the fold values of DNA-binding activity in the CK2 + TBB group compared with the CK2 group. *P < 0.05 vs. control; ^†^ *P* < 0.05 vs. CK2 alone; *n* = 10/group. Values are means ± SDs

Adding CK2 inhibitor TBB after the transfection of CK2 into either H9c2 cells or rat primary ventricular cells produced a marked inhibition of CK2 activity (Fig. 3a). Kir2.1 protein expression is significantly downregulated compared with the sham-treated control cells after the transfection of CK2 . And this repression was efficiently rescued by suppressing CK2 activity with TBB (100 μM) (Fig. 3a). *KCNJ2* mRNA expression was also decreased by CK2 (Fig. 3a). We subsequently verified the effects of CK2 at the functional level. *IK1* was determined in neonatal rat ventricular cells using whole-cell patch-clamp techniques. The cells transfected with CK2 had a lower *IK1* density than the control cells, and the difference was eliminated by adding TBB or valsartan (Fig. 3b). The regulation of the *KCNJ2* gene by CK2 was confirmed via an EMSA, which indicated that CK2 phosphorylates Sp1 to suppress KCNJ2 expression, and the CK2 inhibitor, TBB, eliminates this effect (Fig. 3c).

### Valsartan Inhibits CK2 to Protect KCNJ2/Kir2.1 Following MI

**Fig. 4:**
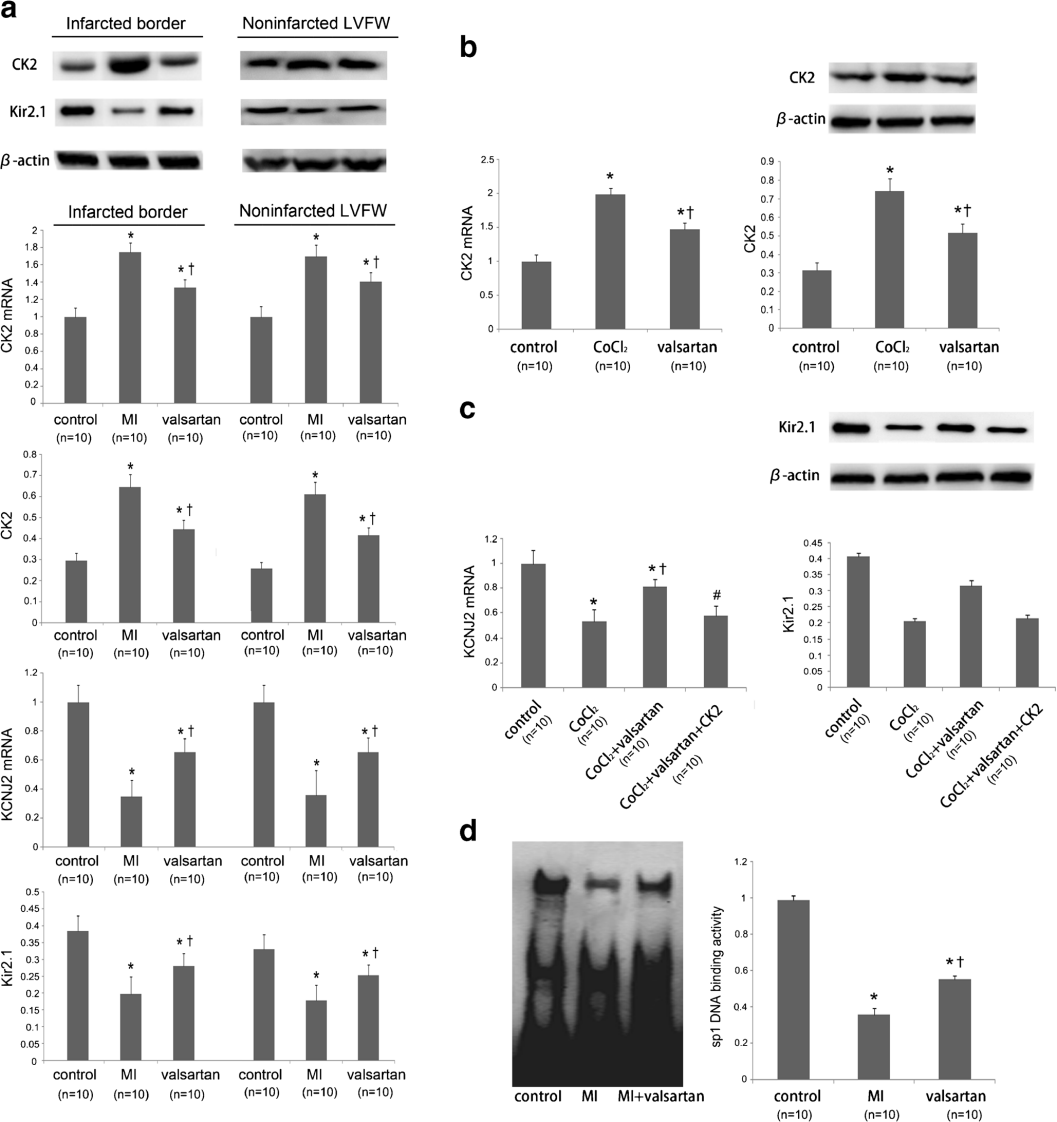
Valsartan inhibited CK2 and protected KCNJ2/Kir2.1. **a** qPCR and immunoblots depicting the effect of valsartan on both CK2 and Kir2.1 in infarcted border and noninfarcted LVFW in MI rats. Both the upregulation of CK2 and the downregulation of Kir2.1 following MI were reversed by valsartan. *P < 0.05 vs. control; ^†^ *P* < 0.05 vs. MI; *n* = 10/group. **b** qPCR and immunoblots depicting the effect on CK2 in H9c2 cells. The upregulation of CK2 by hypoxia induced by CoCl_2_ was depressed by valsartan. *P < 0.05 vs. control; ^†^ *P* < 0.05 vs. CoCl_2_; *n* = 10/group. **c** qPCR and immunoblots depicting the effect on Kir2.1. The downregulation of Kir2.1 by hypoxia was improved by valsartan. Additionally, the over-expression of CK2 in the cells treated with valsartan abrogated this effect. *P < 0.05 vs. control; ^†^ *P* < 0.05 vs. CoCl_2_; ^#^ *P* < 0.05 vs. CoCl_2_ + valsartan; *n* = 10/group. **d** Autoradiograms and the EMSA quantification of Sp1 DNA-binding activity in rat hearts. The data are the fold values of DNA-binding activity in the MI+valsartan group compared with the MI group. *P < 0.05 vs. control; ^†^ *P* < 0.05 vs. CK2; *n* = 10/group. Values are means ± SDs

To determine whether valsartan treatment inhibited electrical remodeling following MI, we tested the expression of CK2 and KCNJ2/Kir2.1. In vivo, the upregulation of CK2 and the downregulation of KCNJ2/Kir2.1 following MI were reversed by valsartan, indicating that valsartan inhibited CK2 to reduce Kir2.1 remodeling following MI (Fig. 4a). In vitro, hypoxia increased CK2 expression, and valsartan reduces the increased CK2 expression induced by CoCl_2_ (Fig. 4b). The over-expression of CK2 in cells treated with valsartan abrogated its beneficial effect on KCNJ2/Kir2.1 (Fig. 4c). Additionally, as the EMSA results indicate, valsartan eliminated the phosphorylation effect of CK2 on Sp1, resulting in a higher *KCNJ2* expression level than in the CK2 group (Fig. 4d).

### Valsartan has Insignificant Effects on Kir2.1 Expression Without Active CK2

**Fig. 5:**
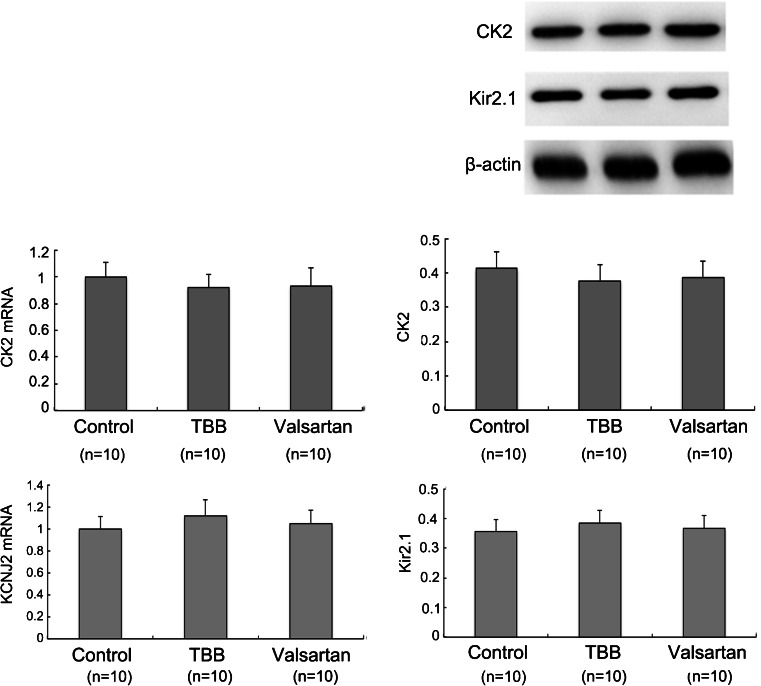
TBB and valsartan have insignificant inhibition effect on the endogenous CK2 as well as Kir2.1 expression. qPCR and immunoblots depicting the effect of TBB and valsartan on CK2 and Kir2.1 in H9c2 cells. Each group had no statistical significance. *n* = 10/group. Values are means ± SDs

To identify whether TBB and valsartan have an effect on Kir2.1 expression through the endogenous CK2, we introduced TBB and valsartan on H9c2 rat ventricular cells without CK2 intervention. Both TBB and valsartan have insignificant inhibitory effects on the endogenous CK2 as well as Kir2.1 expression (Fig. 5). This phenomenon further indicated that valsartan improves KCNJ2/Kir2.1 mostly depending on activated CK2 after MI. While under physiological conditions endogenous CK2 has low activity, leading to weak regulation on Kir2.1 expression.

## Discussion

Taken together, our results indicate that CK2 is a potentially important regulator of *KCNJ2* gene expression and an important determinant of cardiac electronic instability following MI, via *IK1*. Additionally, our findings indicate that CK2 is a potential mediator of the electrophysiological effects of valsartan and provide a basis for the improvement of *IK1* remolding facilitated by valsartan. Therefore, our study has revealed what we believe to be a novel molecular control mechanism of ion channel remodeling following MI.

Cardiac *IK1* current is a strong inward rectifying K^+^ selective current and plays an important role in shaping normal cellular action potentials [30]. Cardiac *IK1* stabilizes the cellular resting membrane potential and is responsible for shaping both the initial depolarization and the final repolarization of the action potential [31, 32]. Studies indicate that *IK1* plays a role in ventricular arrhythmias, as illustrated by the recently described Andersen’s syndrome and studies utilizing guinea pig heart models of ventricular fibrillation [33]. Most of the research regarding the potential role of CK2 in cardiac pathophysiology has been focused on cardiac hypertrophy but has rarely focused on ion channel remodeling. Our findings indicate that CK2 regulates this important K+ channel under specific disease conditions. Further studies regarding the CK2-mediated dysregulation of *IK1* in other pathological contexts may be of interest.

AT1 receptor blockers prevent ventricular dilation, dysfunction, and cardiac hypertrophy in non-infarcted myocardial tissue following MI. Some of the effects observed in patients in the setting of baseline use of RAAS inhibition are related to decreased electrical irritability, which is due primarily to the well-established effects exerted by ARBs on remodeling and the preservation of LV systolic function [34, 35]. Our study showed that valsartan ameliorates KCNJ2/Kir2.1 remodeling during the healing phase after MI when significant structural remodeling also accurs.

CK2 (formerly casein kinase II or CKII) is a ubiquitous protein Ser/Thr kinase with a heterotetrameric structure consisting of two catalytic subunits (42 kDa α and 38 kDa α′) and two regulatory subunits (28 kDa β). CK2 phosphorylates a large number of substrates with various functions related to cell growth and proliferation. However, its electrophysiological effects have seldom been explored. In this study, we firstly investigated CK2 and Kir2.1 mRNA and protein levels in infarcted border and noninfarcted LVFW (left ventricular free wall) seperately to eliminate the different effects of cell necrosis and myocardial remodeling degrees on the levels of mRNA and protein expression. We found that CK2 was activated in vivo following MI and in vitro after the cells were induced to be hypoxia, which resulted in decreased Sp1 DNA binding activity. As a result, the expression of KCNJ2, the flow gene of SP1, was downregulated, as was the expression of Kir2.1 and IK1 current. This effect was repressed by the highly selective cell permeable CK2 inhibitor, TBB, and valsartan. TBB blocks in vitro CK2 activation under hypoxia condition. But as shown in Fig. 5, TBB has little effect on endogenous CK2 in non-hypoxia cells. Valsartan presents a similar effect on CK2 regulation. Without active CK2, both TBB and valsartan have a weak effect on Kir2.1 protein expression. This indicates that CK2 is the main factor mediating the regulation of valsartan on Kir2.1 during the healing phase after AMI.

The mechanism of valsartan inhibiting the actions of CK2 might have two explanations. On the one hand, CK2 might be one downstream protein of AT1 receptor. On the other hand, valsartan has off target effects. Interestingly, CK2 may be activated by AT1 and AT2-activated SHP-1 inactivates CK2 [36]. Indeed, cytoplasmic CK2-alpha’-dependent kinase activity is induced by angiotensin II [37]. Therefore, CK2 seems to be activated by AT1 and inactivated by AT2. After MI, both AT1 and AT2 receptor levels increase. But the degree of upregulated AT1 is higher [38]. Besides, angiotensin II has higher affinity to AT1 than to AT2 [39]. This may explain why CK2 increased after MI in our study and valsartan is efficient in reducing its level.

In conclusion, we have discovered that CK2 regulates the *KCNJ2* gene and its encoded channel, *IK1*. Moreover, valsartan regulates CK2 to improve cardiac ion channel remodeling following MI. But this regulation path still remains to be shown using genetic models. Besides, the improvement of valsartan to *IK1* remodeling may contribute to reduced susceptibility to ventricular arrhythmias during the healing phase of MI and this hypothesis needs to be further demonstrated in vivo. These are two major limitations of our experiment. These findings have provided us with new insights into the molecular mechanisms underlying the cardiac electrical instability that occurs following MI and may represent a treatment strategy for other conditions in which *IK1* is dysregulated.
